# Maintenance of head and neck tumor on-chip: gateway to personalized treatment?

**DOI:** 10.4155/fsoa-2016-0089

**Published:** 2017-03-07

**Authors:** Ruth Bower, Victoria L Green, Elena Kuvshinova, Dmitriy Kuvshinov, Laszlo Karsai, Stephen T Crank, Nicholas D Stafford, John Greenman

**Affiliations:** 1 School of Life Sciences, The University of Hull, Cottingham Road, Hull, HU6 7RX, UK; 2 Department of Chemical & Biological Engineering, The University of Sheffield, Mappin Street, Sheffield, S1 3JD, UK; 3 School of Engineering & Computer Science, The University of Hull, Cottingham Road, Hull, HU6 7RX, UK; 4 Department of Cellular Pathology, Hull Royal Infirmary, Anlaby Road, Hull, HU3 2JZ, UK; 5 Department of Oral & Maxillofacial Surgery, Hull Royal Infirmary, Anlaby Road, Hull, HU3 2JZ, UK; 6 Castle Hill Hospital, University of Hull, Daisy Building, Cottingham, HU16 5JQ, UK

**Keywords:** head and neck cancer, microfluidic, tissue culture, tumor

## Abstract

**Aim:**

Head and neck squamous cell carcinomas (HNSCC) are solid tumors with low overall survival (40–60%). In a move toward personalized medicine, maintenance of tumor biopsies in microfluidic tissue culture devices is being developed.

**Methodology/results:**

HNSCC (n = 15) was dissected (5–10 mg) and either analyzed immediately or cultured in a microfluidic device (37°C) for 48 h. No difference was observed in morphology between pre- and postculture specimens. Dissociated samples were analyzed using trypan blue exclusion (viability), propidium iodide flow cytometry (death) and MTS assay (proliferation) with no significant difference observed highlighting tissue maintenance. Computational fluid dynamics showed laminar flow within the system.

**Conclusion:**

The microfluidic culture system successfully maintained HNSCC for 48 h, the culture system will allow testing of different treatment modalities with response monitoring.

## Lay abstract

Head and neck cancers often have a poor treatment outcome. In order to study the response of the tissue, a miniaturized culture system has been developed to keep a small piece of tumor alive. In the current study, we show that small pieces of cancer tissue can be maintained in the system, using tissue structure and viability of single cells as a guide. In future work, patient equivalent treatments can be applied to these microculture systems to investigate individual patient tumor responses, which could help to guide treatment selection.

Head and neck cancers are a diverse group of malignancies arising from cells within the mucosal surfaces of the oral cavity, pharynx, larynx, salivary glands and nasal cavities [1] and are the sixth most common solid tumor worldwide [2] with squamous cell carcinomas (head and neck squamous cell carcinomas [HNSCC]) constituting 90% [3]. A significant proportion of patients present with regional nodal involvement (40%) and a further 10% present with the additional complication of distant metastasis [4]. Chemoradiation is the predominant primary treatment, but despite multiple advances over recent years, such as the introduction of intensity-modulated radiotherapy [5,6], treatment resistance is a persistent problem, contributing to the low overall 5-year survival figures of 40–60% for patients treated with radiotherapy or chemoradiotherapy [7].

As a result, the desire for targeted approaches to treatment has intensified [8,9]. However, to date cetuximab (the anti-EGFR antibody) is the only approved targeted therapy for the treatment of recurrent metastatic head and neck disease [10]. Median duration of locoregional control was extended by 9.5 months with cetuximab plus radiotherapy compared with radiotherapy alone [11]. In addition, searches for biomarkers to aid treatment selection have proved challenging with no standard-of-care biomarkers currently in use in all centers [12].

The human papilloma virus (HPV) is an important etiological agent in some subsets of HNSCC, with HPV-positive cancers having been shown to have a better prognosis and response to therapy than their HPV-negative counterparts [13,14]. HPV status is the strongest predictor of overall and disease-specific survival, as well as locoregional control [15]. Therefore, in a few centers, high-risk HPV variants are initially screened for positivity by p16 immunohistochemistry followed by PCR and/or *in situ* hybridization, in order to direct Phase III treatment de-escalation trials [4]. Beyond this, no technologies or screening methodologies to personalize therapy specifically to individual patients exist.

A large range of model systems exist to study tumor biology and treatment response including cell culture, spheroids, organoids and mouse models. While these methodologies have facilitated advancement of the field, they also have associated pitfalls; the use of a single cell type or even multiple cell types underestimates the complex nature of the *in vivo* microenvironment comprising numerous cell types in a unique spatial arrangement within an extracellular matrix [16]. Three-dimensional culture aims to recapitulate the spatial arrangement of cells yet still lacks complexity, including the full gamut of cell types such as tumor-infiltrating lymphocytes [17]. Xenograft models provide continuous flow to engrafted tumors within an *in vivo* environment, however, procedures are costly and lengthy and have clear ethical considerations [18], particularly at a time when there are calls for a reduction in the use of animals in research.

Microfluidic culture systems involve the continuous perfusion and removal of waste from a tissue sample *ex vivo*, in a fashion mimicking the function of the circulatory system [19]. Multiple pieces from the same initial tissue sample can be perfused in parallel devices allowing the study of a range of variables with replicates. This provides a potential low-cost method for investigating the biology of patient samples and response to therapeutic assault in a personalized fashion.

Microfluidics has been defined as ‘*the science and technology of systems that process or manipulate small (10^-9^–10^-18^ liters) amounts of fluids, using channels with dimensions of tens to hundreds of micrometres*’ [20]. The small channel geometries afford the benefit of laminar flow within devices allowing a high level of spatial control. Laminar flow is defined at Reynolds numbers (Equation 1) below 2000 [21].
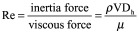



Equation 1: Equation used to generate the Reynolds number within a microfluidic device, where *ρ* (kg/m^3^) is fluid density, *µ* is dynamic viscosity of fluid (kg/m/s), *D*_h_ (m) is the hydraulic diameter and *V* (m/s) is velocity.

Fluid flow modeling of devices by computational fluid dynamics (CFD) can afford benefits in the visualization of the properties discussed above, allowing manipulation of designs to achieve the closest possible resemblance of the *in vivo* environment for the biomedical model. CFD application in biomedical devices is relatively recent with more traditional applications being found in chemical and mechanical engineering [22].

The dawn of microfluidic applications in tissue culture began in 2003 with the culture of rat brain tissue slices [23], more recently (since 2013), various devices have been developed for the culture of human (Table 1) and animal tissue [24–28] with the potential of monitoring the response to therapeutic assault or external stimuli.

**Table 1.** **Published literature on the use of microfluidics for *ex vivo* tissue culture from human samples (1 January 2013 to 1 November 2016).**

**Tissue**	**Summary of work conducted**	**Ref.**
Intestinal sections (small or large bowel)	A dual-flow microfluidic device maintained full thickness intestinal tissue in known orientation for up to 3 days. The device perfused luminal and serosal sides independently. Tissue maintenance was demonstrated by LDH release, H&E and Ki67 staining. ELISAs were conducted on system effluents for Calprotein (s100a8; inflammatory marker)	[30]
Prostate and ovarian cancer xenograft and primary serous ovarian cancer	‘Spheroid-sized’ MDTs (380 µM diameter and 300 µM height) were maintained in a microfluidic device with regular medium replacement for up to 8 days. Viability was determined on chip by confocal microscopy and off-chip by flow cytometry on dissociated cells (annexin V and 7AAD, xenograft only). A proof of concept experiment showed carboplatin chemosensitivity testing on a primary ovarian cancer tissue	[31]
Human adipose tissue	3-mm diameter hAT biopsies maintained for up to 7 days were used to study insulin resistance in Type 2 diabetes mellitus patients compared with healthy controls. Viability was evaluated by MTT assay and morphology shown by off-chip H&E staining. Glucose uptake was used as a measure of insulin resistance	[32]
HNSCC	Culture of HNSCC biopsies (5–10 mg) for 6 days was used to analyze the response of tissue to irradiation (2–40 Gy). Cell death was measured by detection of LDH in the effluent, and immunohistochemistry for cleaved cytokeratin-18 was used to calculate an apoptotic index	[33]
Prepuce and occipital and temporal scalp skin follicular unit extracts	Culture device was developed to prolong culture period of skin biopsies and skin equivalents. Punched biopsies (5 mm) of prepuce and *ex vivo* FUEs were cultured separately in the device for 14 days. Samples were taken from the device and cryosectioned for downstream analysis of morphology (H&E staining) and immunofluorescence for proliferation (Ki67) and apoptosis (TUNEL)	[34]
HNSCC	HNSCC biopsy tissue (5–10 mg) maintained for up to 9 days was used to allow testing of multiple chemotherapy agents (Cisplatin, Fluorouracil and Docetaxel) alone and in combination. Response was measured in the system effluent; cell death was analyzed by LDH and viability was measured by WST-1 proliferation assay	[35]

7AAD: 7-Aminoactinomycin D; FUE: Follicular unit extract; Gy: Gray; hAT: Human adipose tissue; H&E: Hematoxylin and eosin; HNSCC: Head and neck squamous cell carcinoma; LDH: Lactate dehydrogenase; MDT: Microdissected tissue; MF: Microfluidic; MTT: 3-(4,5-dimethylthiazol-2-yl)-2,5-diphenyltetrazolium bromide; TUNEL: Terminal deoxynucleotidyl transferase dUTP nick-end labeling; WST: Water-soluble tetrazolium salt.

The capability of monitoring tissue response on an individual basis using microfluidic technology hopes to address the clinical problem of treatment resistance (Figure 1). The field has received some scepticism regarding the use of these devices for tissue maintenance with questions arising over sample viability and the relevance of the data obtained to the *in vivo* environment [29].

**Figure 1.**
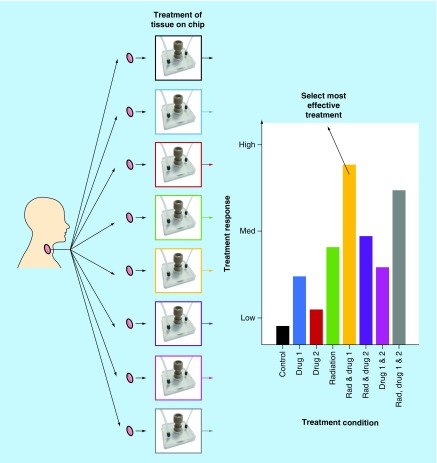
**Schematic diagram showing the parallel nature of the on-chip treatment testing concept.** *Ex vivo* testing of tissue response provides potential for the selection of treatment on an individual patient basis.

The aim of the current study was to address the uncertainty within this exciting field by generating sound evidence for the maintenance of malignant tissue within the microfluidic culture device, without loss of sample viability. Herein, flow cytometry is used to determine cell death within HNSCC samples before and after microfluidic culture; this is the first time these techniques have been used in conjunction to assess primary patient tissue. In addition, the in-house-designed microfluidic culture device was modeled for fluid flow dynamics.

## Materials & methods

### Patient biopsies

Tumor tissue samples (n = 15) were obtained from patients with HNSCC in accordance with the Local Research Ethics Committee (LREC-10/H1304/6) and NHS R&D approval (R0897), following written informed consent. Patients with no history of previous treatment undergoing resection surgery, to remove either laryngeal, oropharyngeal or oral cavity tumors staged at T2–T4 were recruited; associated metastatic lymph nodes staged at N1–N2 were also collected where possible. Fresh tissue samples were processed and cultured within 90 min of excision.

### Microfluidic device & culture system

**Figure 2.**
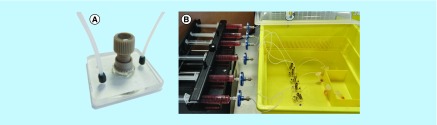
**Photographs of microfluidic culture device and setup.** **(A)** Device close up including PEEK plastic microport and PDMS-filled PEEK-threaded adaptor. Connected to inlet and outlet ETFE tubing via graphite ferrules. **(B)** Complete culture setup with syringes containing complete medium attached to a pressure-driven syringe pump for continuous flow to the tissue sample within the device. Effluent was collected in Eppendorf tubes and the devices were maintained in an incubator at 37°C. ETFE: Ethylene tetrafluoroethylene; PDMS: Polydimethylsiloxane; PEEK: Polyether ether ketone.

The microfluidic device was fabricated from two layers of thermally bonded B270 glass in the Department of Chemistry at the University of Hull (Figure 2A), the upper layer was 3 mm in depth and lower glass layer 1 mm. One inlet hole, one outlet hole and the central tissue chamber were drilled into the upper layer corresponding to channels (170 µm wide and 70 µm deep) in the lower glass layer, fabricated by photolithography and wet etching techniques [36]. Over the central well, a PEEK plastic microport was bonded with epoxy adhesive, into which an English threaded adaptor (both IDEX Kinesis, Cambridgeshire, UK), filled with polydimethylsiloxane (Dow Corning, Barry, UK), was screwed to seal the chamber.

ETFE tubing (IDEX Kinesis,) with an internal diameter of 0.5 mm was secured to the inlet and outlet hole by epoxy adhesive and a graphite ferrule (Figure 2A; IDEX Kinesis). Dulbecco's Modified Eagles Medium (DMEM; Lonza, Slough, UK) containing 25 mM HEPES (4-(2-Hydroxyethyl)piperazine-1-ethanesulfonic acid) buffer and 4.5 g/l glucose supplemented with 10% [v/v] fetal bovine serum (Labtech, East Sussex, UK), penicillin/streptomycin 0.1 U/ml and 0.1 mg/ml, respectively, 0.1 mM NEAA (nonessential amino acids), 2.5 µg/ml Amphotericin B and 2 mM L-glutamine (All Lonza; hereafter referred to as complete medium) was connected to the ETFE inlet tubing via a 2-part adapter (Lab smith, CA, USA). A 5–10-mg HNSCC sample was placed into the central tissue chamber and the system was connected to a Harvard PhD 2000 syringe pump (Harvard, UK), the device and tubing were maintained at 37°C inside an incubator with pressure-driven flow of complete medium at a volumetric flow rate of 2 µl/min (Figure 2B). Each device was run for 48 h with effluent collected in 1.5-ml Eppendorf tubes.

### Frozen tissue embedding & morphological analyses

Tumor sample was embedded in Tissue-Tek OCT (Optimum Cutting Temperature; Sakura, Berkshire, UK), by freezing in liquid nitrogen-cooled 2-methyl butane (Sigma-Aldrich, Dorset, UK) both prior to and following microfluidic culture. Tissue sections of 8 µM were cut on a Leica CM1100 Cryostat and fixed for 20 min in -20°C cooled methanol, stained in Harris’ Hematoxylin (Sigma-Aldrich) for 5 min before differentiating in 1% acid alcohol (concentrated HCl in 70% Ethanol) and rinsing in running tap water. The tissue was then stained with 1% (w/v) Eosin Y (Sigma-Aldrich) in tap water for 5 min before washing in tap water. The tissue was dehydrated through graded alcohols (70, 90 and 100%) and three changes of histoclear before mounting in histomount. Slides were visualized using light microscopy (Nikon ECLIPSE E800) and photographed (Micropublisher 5.0 Real Time Viewing) utilizing Image-Pro premier software (MediaCybernetics, Cambridge, UK). Detailed observations of the tissue characteristics were carried out under close supervision of a consultant head and neck pathologist, Lazslo Karsai.

### Tissue dissociation

Dissociation of tumor tissue prior to and following microfluidic culture was achieved using a combination of mechanical and enzymatic methods; samples of a known weight were placed into a secured lid of a 1.5-ml Eppendorf tube under aseptic conditions and minced using a criss-cross cutting action. Complete medium (1 ml) containing 0.02% (w/v) Collagenase IV (Sigma-Aldrich) and 0.02% (w/v) DNase I (Roche, Hertfordshire, UK) was added to the Eppendorf tube and the lid containing the minced tissue was replaced before incubation on a rotator for 2 h at 37°C and 5% CO_2_. Following enzyme incubation, the suspension was strained (70-µm cell strainer, BD, Oxford, UK) and centrifuged at 400× *g* for 5 min, the pellet was resuspended in 1 ml of complete medium and percentage viability of tissue determined using Trypan Blue exclusion.

### Propidium iodide flow cytometry

The resultant cell suspension from the dispersal of a single 5–10-mg tissue sample (n = 8) prior to and after microfluidic culture was washed in phosphate-buffered saline (PBS; 5 ml) containing 0.25% (w/v) BSA (Bovine Serum Albumin; Sigma-Aldrich) and 0.06% (w/v) sodium azide (Sigma-Aldrich; PBS/BSA/Azide) and divided between two tubes before centrifugation at 400× *g* for 5 min. Pellets were resuspended in 100-µl PBS/BSA/Azide, and either stained with 500-µg/ml propidium iodide (PI; Sigma-Aldrich) for 15 min in the dark and or remained unstained as a control. Following a further wash, the cells were resuspended in 300-µl PBS/BSA/Azide and analyzed in the FL-3 channel on a flow cytometer (BD FACSCalibur), a minimum of 10,000 events were acquired and a histogram was plotted to determine the percentage of dead cells.

### MTS proliferation assay

Metabolic activity/proliferation of dissociated cells prior to and following microfluidic culture was determined using the Promega CellTiter 96^®^ AQueous One Solution (Promega, Southampton, UK) according to the manufacturer's instructions. Briefly, dissociated cells from a single 5–10-mg tissue were resuspended in 200 µl of complete DMEM. The suspensions were cultured in duplicate overnight in a 96-well flat-bottomed plate before the MTS assay reagent (20 µl) was added to each well for 2 h at 37°C prior to measuring absorbance of the formazan product at 492 nm (Thermo Scientific Multiskan FC, Thermo Scientific, Hampsted, UK). Absorbance values were standardized per milligram of starting weight of tissue.

### Statistics

Statistical analyses were performed using SPSS statistics 23 (IBM, Portsmouth, UK) and p-values <0.05 were considered significant. Differences between the preculture and postculture samples were tested for significance using independent samples’ t-tests.

### Fluid flow modeling

Generally, fluid dynamics is described by governing equations [37]:

Conservation of mass equation:
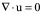



where *u* is flow velocity vector.

Conservation of momentum equation for incompressible fluid:
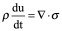



where *ρ* is density, *t* is time, *σ* is total stress:
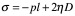



where *p* is pressure, *η* is dynamic viscosity, ***D*** is rate of strain tensor, ***I*** is identical matrix:
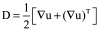



where *^T^* shows the transpose operation.

Computer simulation of pressure-driven Poiseuille flow in the chip was realized using COMSOL software, version 5.2a (COMSOL, Cambridge, UK). The discretization of governing partial differential equations was made by the finite element method. Rheological (flow) parameters of the DMEM medium were assumed to be the same as water (temperature = 37°C). The meshing was performed using the physics-controlled method with normal-sized triangle elements from 0.003 to 0.1 mm, the total number of elements was 14,736. The inlet flow rate was set as an inlet boundary condition, and the volumetric flow rate of 2 μl/min was transformed with regard to the inlet width of 0.5 mm. At the outlet, p = 0 is established.

Navier-Stokes governing equations for incompressible steady-state flow real geometry of the chip were solved using COMSOL Multiphysics modelling software to obtain velocity field and flow pattern for Newtonian fluid. The built-in ‘Laminar flow’ module for Newtonian fluid (constant dynamic viscosity) was selected to simulate fluid flow at given conditions.

## Results

Tumor characteristics of patient biopsies, tumor site, T and N classification and patient age and gender were collected (n = 15; Supplementary Table 1).

### Morphological analyses

**Figure 3.**
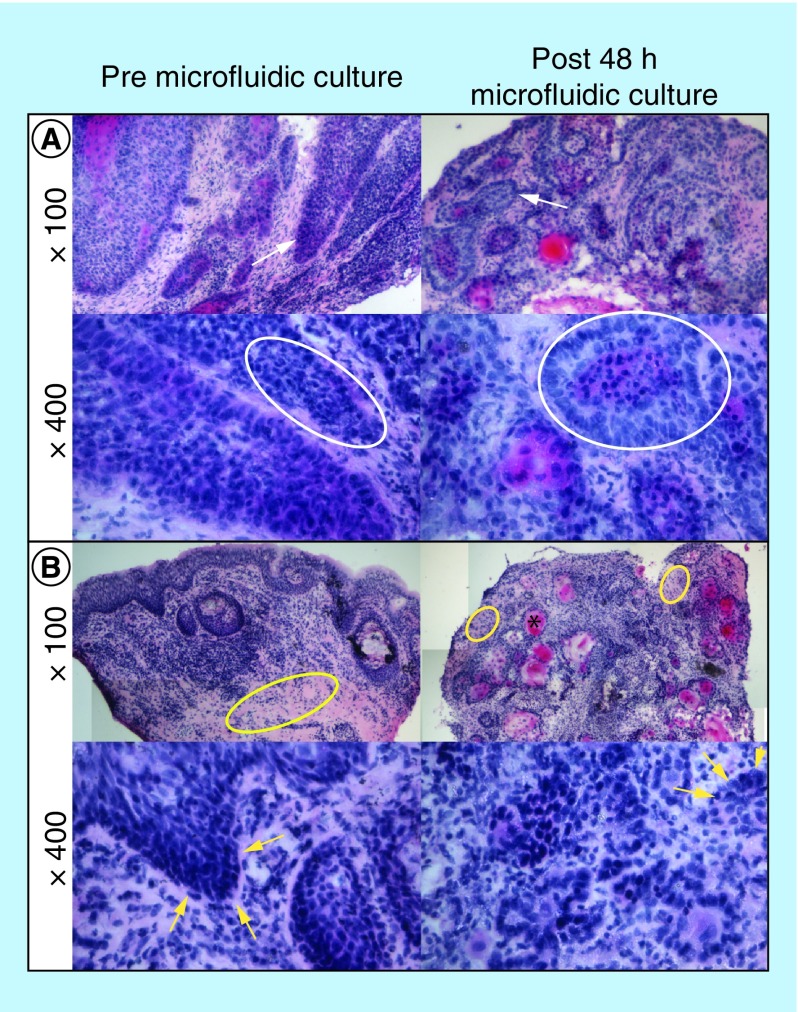
**Hematoxylin and Eosin-stained 8-µm tissue sections, shown at 100*×* and 400*×* magnification.** Two representative tumors are shown (n = 15). **(A)** Laryngeal squamous cell carcinoma, staging T4aN0M0. **(B)** Transglottic laryngeal squamous cell carcinoma, staging T4N0M0. Each tumor is shown pre- and post-48-h microfluidic culture. The bright pink-stained areas on some of the images depict extracellular keratin (keratin pearls*), arising from the squamous cells. White arrows show infiltrating islands and cords of tumor cells. Tumor area showing orientation of cell maturation is circled in white. Pale stromal areas with inflammatory cells are circled in yellow. Intact basement membrane is shown by yellow arrows.

Hematoxylin and eosin (H&E)-stained HNSCC tissue sections were visualized before placement of the tumor in the microfluidic culture device (Figure 3, left panel) and following 48 h of continuous perfusion within the device (Figure 3, right panel).

Representative images are shown from two patients with laryngeal squamous cell carcinoma (A & B). The low-magnification (100×) images show maintenance of the tissue structure. Infiltrating islands and interconnected cords of tumor cells are shown in the premicrofluidic culture images and are retained following 48-h culture. Pale pink stromal areas of the tumor are also apparent before and after microfluidic incubation.

The higher magnification (400×) allows the assessment of cellular characteristics and shows an intact basement membrane following microfluidic culture. Furthermore, the orientation of cell maturation seen in premicrofluidic culture tissue, with more mature cells observed in the center of tumor islands and nests was also present post-48-h microfluidic culture. The chronic inflammatory mononuclear cell fraction (lymphocytes and macrophages) within the stroma was recognizable both before and after microfluidic culture.

### Viability analyses

**Figure 4.**
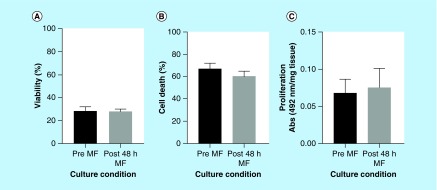
**Viability, cell death and proliferation analysis of single cell suspensions from dispersed tumor tissue before and after microfluidic culture.** **(A)** Percentage viability of cell population by trypan blue exclusion (n = 15). **(B)** Percentage cell death by PI staining and flow cytometry from 10,000 events (n = 8). **(C)** Proliferation (MTS assay) of cell suspensions following overnight culture quantified by absorbance of colored formazan product at 492 nm and normalized per milligram of tissue (n = 7). Mean + SEM. PI: Propidium iodide.

No significant difference in percentage viability between tumor tissue obtained following surgical resection (11–57.9%) and a sample from the same tumor cultured in the microfluidic device (17.1–40.4%) for 48 h was observed using trypan blue exclusion (Figure 4A; n = 15). The viable number of cells obtained per milligram of tissue was also calculated, with tissue dissociated immediately yielding an average of 5.71 × 10^4^ ± 3.14 × 10^4^ viable cells/mg tissue, and that dissociated following 48 h of microfluidic culture yielding 4.36 × 10^4^ ± 1.63 × 10^4^ viable cells/mg tissue, again with no significant difference between the two conditions. For comparison, flow cytometry was utilized to quantify cell death by PI staining; again no significant difference in percentage cell death was observed between the dissociated tumor cells before and after 48-h microfluidic culture (Figure 4B; n = 8). The viability (by trypan blue) and cell death (as measured by PI staining) results were concordant.

### Tumor cell proliferation

A variation in proliferative capacity of the dissociated tumor cells was observed between samples with formazan product absorbance (492 nm) values ranging from 0.026 to 0.140 in preculture samples and that from post-48-h culture samples being 0.022–0.164 (per milligram of tissue). Consequently, no significant difference in proliferation of the cell suspensions was observed between pre- and post-48-h culture (Figure 4C; n = 7).

### Device modeling

The experimental cell configuration is not axisymmetric, but symmetric along the central plane. This plane, which crosses the input–output channel along the middle line, is the most information-rich area, and provides the flow pattern, characterized by highest reynolds number variability. Thus, based on the assumption, that further developing of the model to 3D would not contribute significantly to the flow profile investigation, and would greatly increase simulation time, the decision was made to apply the 2D model to the study. This model allows investigation of the main issue of the study: the flow uniformity.

**Figure 5.**
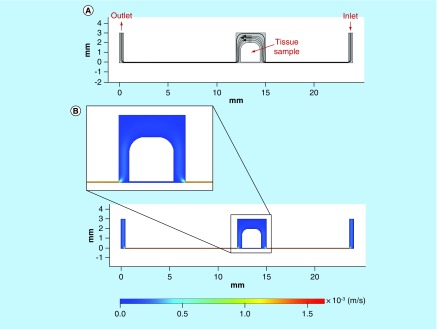
**Computer simulation of fluid flow and velocity field in the microfluidic culture device.** **(A)** Streamlines and velocity vectors of laminar fluid flow in the device. Labels show inlet, outlet and tissue sample location. **(B)** Velocity magnitude of the flow within the device. Color legend explains value of the velocity along the channel and the treatment chamber (m/s); thus, the maximum of the velocity equals 1.5 × 10^-3^ m/s or 1.5 mm/s.

In the first instance, the ‘perfect’ flow simulation was modeled that is where the placement of the tissue sample within the device was central with no deformation to the shape of the tissue apparent as a result of the force exerted by the flow (Figure 5).

**Figure 6.**
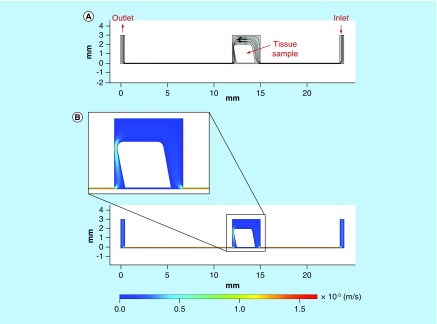
**Computer simulation of fluid flow in the microfluidic tissue culture device, with tissue sample tilted 10° due to the flow pressure.** **(A)** Streamlines and velocity vectors of laminar fluid flow in the device. Labels show inlet, outlet and tissue sample location. **(B)** Velocity magnitude of the flow within the device. Color legend explains value of the velocity along the channel and the treatment chamber (m/s); thus, the maximum of the velocity equals 1.5 × 10^-3^ m/s or 1.5 mm/s.

As the structure, shape and the placement of the sample within the chamber of the device could all be changed as a result of the flow forces acting on the tissue. The device was modeled with a tilt of 5 and 10° to show the change to the fluid flow in this situation (Figure 6; 10° model).

**Figure 7.**
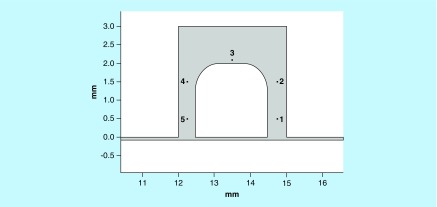
**Comparative analysis of the velocity magnitude and Reynolds number of the flow along the sample.** Point placement along the sample for further analysis.

In order to quantify the flow characteristics, numerically five points around the tissue were chosen for further study (Figure 7A). Point 3 was placed 0.1 mm away from the tissue boundary while points 1, 2, 4 and 5 were placed 0.25 mm away from the tissue boundary in all tilt conditions (except in the case of point 4 in the 10° tilt analysis where the distance was 0.09 mm). Calculated values for Reynolds number and velocity magnitude are shown (Table 2).

**Table 2.** **Velocity magnitude of fluid flow around the sample with different displacement angles (0, 5 and 10°) and Reynolds number within the device.**

**Angle of tilt (degrees)**	**Velocity magnitude (mm/s)**				
	**Point 1**	**Point 2**	**Point 3**	**Point 4**	**Point 5**
0	0.2	0.19	0.04	0.24	0.24
5	0.19	0.17	0.039	0.26	0.26
10	0.16	0.12	0.039	0.64	0.3
**Reynolds number**					
0	1.9 × 10^-3^	1.3 × 10^-3^	4.4 × 10^-4^	1.5 × 10^-3^	2.2 × 10^-3^
5	9.4 × 10^-4^	6.0 × 10^-4^	1.4 × 10^-4^	1.0 × 10^-3^	1.4 × 10^-3^
10	1.6 × 10^-3^	1.3 × 10^-3^	3.4 × 10^-4^	5.1 × 10^-3^	3.1 × 10^-3^

Point locations are shown Figure 7.

The Reynolds numbers obtained show values’ characteristic of laminar flow at the 0, 5 and 10° tilt conditions. Velocity magnitude data shows key differences within the fluid flow at point 4 in the tilted 10° condition, given the reduced distance between the tissue and the chamber wall the velocity magnitude was increased by over 2.5× compared with the 0° initial, no tilt condition. Furthermore, the velocity magnitude at point 4 was 5.3× higher than that at point 2 in the 10° tilt condition, whereas in the 0° tilt condition point 4 velocity was only 1.26× higher than point 2. Differences in velocity at all other points were minimal.

## Discussion

The biological data yielded promising results in terms of viability maintenance following microfluidic culture; intact morphology following microfluidic culture was demonstrated by H&E staining with the presence of distinct characteristics of squamous cell carcinoma. This is in agreement with previous studies from the group, using both rat liver and HNSCC tissue [38,39] and those of others who used H&E to demonstrate maintenance of tissue-specific structures including single hair follicular units and human adipose tissue [32,34] with culture times in microfluidic devices ranging from 1 to 14 days.

The use of enzymatic and mechanical tissue dissociation to obtain a single cell suspension for subsequent analysis allows a more precise determination of cell viability and increases the scope of analytical methods that are achievable; however, it may also increase the chance of experimental-induced damage. The results obtained from single cell suspensions showed concordance between trypan blue exclusion and PI flow cytometry. Previously, the use of flow cytometry following microfluidic culture has received little attention and has only been utilized on prostate and ovarian cancer xenograft tissues [31]. In that study, the maintenance of small microdissected tissues (MDTs; 380 µM diameter and 300 µM height) was maintained in a microfluidic device with periodic media changes, continuous perfusion was not employed. MDTs were stained with annexin V and 7AAD prior to dissociation (0.25 mg/ml collagenase IV in saline 15 min at 37°C) followed by flow cytometric analysis. A minimum of 40% viability (shown by unstained cells) for 8 days across four different xenografts was obtained. Results presented in the current study, using PI to assess cell death, detail the first flow cytometry data on human primary tissue following microfluidic culture, and showed no increase in cell death following the 48-h culture period, although the data reveal lower viabilities (preculture average: 28.9%, post-48-h culture average: 28.1%) than the 40% reported previously [31]. This could be attributable to the difference in starting material (xenograft vs primary tissue) and the experimental procedure differences, particularly staining the tissue with flow cytometry markers before or after dissociation. Other work maintaining HNSCC in microfluidic devices showed no significant difference in apoptotic cell death (apoptotic index, m30 immunohistochemistry [IHC] staining) between preculture and post-4-day culture, again indicating maintenance of tissue [33]; however, samples were not disaggregated for cellular analysis.

The range of viable cells/mg obtained experimentally from HNSCC tumors before (1.5 × 10^4^–1.4 × 10^5^) and after 48-h microfluidic culture (1.9–7.6 × 10^4^) demonstrated no significant effect of microfluidic culture on the number of viable cells within the tumor tissue. Trypan blue exclusion has previously been used following combined mechanical and enzymatic dissociation methods (tissue minced, incubated 4°C with 0.5% trypsin 12–20 h, then agitated with rotating razor blades for 5–15 min) on solid tumors’ (small-cell lung carcinomas and testicular cancer) preculture yielding 1.5–5 × 10^4^ viable cells/mg [40], thus our experimental system appears to extend the range from that previously reported.

The current study showed no significant difference between the average proliferation of HNSCC samples preculture and post-48-h microfluidic culture by the MTS proliferation assay, as the variance in the results observed was high (0.026–0.146 and 0.022–0.164, respectively, absorbance_492nm_/mg tissue). The range in proliferation is a relevant observation from the data and correlates with the known intertumor heterogeneity affecting key cancer pathways driving phenotypic variation [41]. Proliferation of HNSCC is known to vary between anatomical subsites, for example, Pedicini *et al*., correlated EGFR expression of patient tumors to HNSCC subsites with high-EGFR expression associated with greater proliferation following irradiation [42]. Further variation is also seen within tumors, (intraheterogeneity: [41,43,44]) which could in part explain the variation seen between the preculture and post-48-h microfluidic culture conditions. The use of other metabolic activity assays, similar to MTS, as a surrogate for proliferation has been used by others (MTT assay); to compare human adipose tissue before and after 7 days of culture in a microfluidic device again with no difference in absorbance observed between the preculture and following microfluidic culture conditions [32].

Maintenance of patient tissue on a chip is an emerging technology with cell culture or ‘engineered tissue’ culture on a chip more widely employed; in part due to the relative simplicity of sample acquisition and availability. However, the use of cell lines, spheroids and engineered tissue will always simplify the clinical situation and as such often extend culture periods. For example, spheroids created from a breast cancer cell line or a breast cancer cell line coculture with a fibroblast cell line were maintained for up to 14 days shown using Calcein AM (AM; acetoxymethyl ester group) as a viability marker [48]. Utilizing primary tissue samples as an alternative holds key benefits in the translational relevance and application of the data obtained from the devices to real-world problems, such as treatment resistance.

The use of modeling to better understand the flow patterns within microfluidic devices can aid understanding and interpretation of experimental observations and results; this extra dimension provides information regarding the surrounding environment of the tissue sample. Furthermore, the modeling can be used as a tool to improve the design of devices in order to enhance the functionality of the device and/or mimic the *in vivo* situation. The Reynolds number obtained for the device in the current study indicates that the flow within the channels is ‘creeping flow’ also known as the Stokes law region (Reynolds number <1 [49]), where the viscous forces of the fluid dominate over the inertial forces, due to the low fluid velocities [50]. Thus, the flow within the device can be classified as laminar, a key property in the employment of microfluidic devices in biomedical applications.

Although this laminar flow in the current device has been utilized successfully to maintain HNSCC tissue, the modeling has identified some areas for improvement, namely the stagnant flow areas around the base of the tissue within the chamber; these areas result in reduced perfusion of the tissue. Moreover, it could not be excluded that at higher mass inflow the sample would not touch the chamber walls due to flow pressure, so this positioning of the tissue would form a turbulent flow pattern with vorticities and zones with velocities close to zero. Device modeling has been utilized previously to develop designs that allow manipulation of different experimental conditions; for example, mathematical modeling described fluid dynamics alongside intracellular signaling as a key predictor in glucose uptake in adipose tissue [32] and assessment of oxygen delivery to tumor samples within a microfluidic device [31].

## Conclusion

Microfluidic culture successfully maintains HNSCC samples for 48 h such that they are comparable to the preculture sample in terms of morphology and viability with expected variation between patients. The study provides scope for extending this time period in future work. The current microfluidic device design facilitated laminar flow, however, the design also resulted in some ‘dead zones’ where the fluid velocity was virtually nonexistent resulting in limited perfusion. Despite this, the maintenance of the tissue viability within the microfluidic device is encouraging and invites subsequent studies applying treatment regimens to HNSCC samples within optimized on-chip devices.

## Future perspective

Treatment resistance is a persistent problem yielding a worse prognosis and reducing clinical success for patients [51]. The selection of a treatment for an individual patient based on the responsiveness of their malignancy to therapeutic assault could customize care and improve outcomes. Culture of small HNSCC samples using microfluidic culture as described or in fact the culture of any solid tumor could facilitate such testing, including that of novel agents. Additionally, modifications in device design guided by CFD could improve perfusion to the whole surface of the tissue and as such increase the length of the culture period. Future work on treatment application to these devices will increase the translational relevance of the technology allowing monitoring of patient-specific tumors and their response to therapeutic assault, in order to direct treatment decision-making.

**Executive summary**

- Head and Neck Squamous Cell Carcinomascan be maintained in our tumor-on-chip device without loss of sample viability for 48 h.
- Squamous cell carcinoma tissue morphology characteristics are maintained following culture.
- Computational fluid dynamics alongside biological data is valuable in terms of characterizing the sample environment and improving the model system.
- Establishment of an optimized, defined, model system will allow tissue to be tested for response to different treatment modalities simultaneously.
