# Supplementary material for: Maintenance of head and neck tumor on-chip: gateway to personalized treatment?
Source: Future Sci OA. 2017 Mar 7;3(2):FSO174. doi: 10.4155/fsoa-2016-0089 (PMC5481812; doi:10.4155/fsoa-2016-0089)
Supplement: Supplementary file 1 [file fsoa-03-174-s1.docx]

| **Supplementary table 1.** Tumour characteristics of HNSCC patient biopsies. | |
| --- | --- |
| ***Characteristic*** | ***Number of tumours*** |
| **Tumour site:**  Larynx  Oropharynx  Oral cavity  Cervical node disease | 4  3  5  3 |
| **T classification**  T1  T2  T3  T4  Unknown | 0  1  4  8  2 |
| **N Classification**  N0  N1  N2  Unknown | 7  1  4  3 |
| **M Classification**  M0  M1 | 15  0 |
